# Epigenetic silencing of tumor suppressor *miR-3151* contributes to Chinese chronic lymphocytic leukemia by constitutive activation of MADD/ERK and PIK3R2/AKT signaling pathways

**DOI:** 10.18632/oncotarget.6251

**Published:** 2015-10-27

**Authors:** Lu Qian Wang, Kwan Yeung Wong, Anders Rosèn, Chor Sang Chim

**Affiliations:** ^1^ Department of Medicine, Queen Mary Hospital, The University of Hong Kong, Hong Kong; ^2^ Department of Clinical and Experimental Medicine, Linköping University, Linköping, Sweden

**Keywords:** microRNA, miR-3151, tumor suppressor, DNA methylation, chronic lymphocytic leukemia

## Abstract

We hypothesize that *miR-3151*, localized to a GWAS-identified chronic lymphocytic leukemia (CLL) risk locus (8q22.3), is a tumor suppressor miRNA silenced by promoter DNA methylation in CLL. The promoter of *miR-3151* was methylated in 5/7 (71%) CLL cell lines, 30/98 (31%) diagnostic primary samples, but not normal controls. Methylation of *miR-3151* correlated inversely with expression. Treatment with 5-Aza-2′-deoxycytidine led to promoter demethylation and *miR-3151* re-expression. Luciferase assay confirmed MAP-kinase activating death domain (MADD) and phosphoinositide-3-kinase, regulatory subunit 2 (PIK3R2) as direct targets of *miR-3151*. Moreover, restoration of *miR-3151* resulted in inhibition of cellular proliferation and enhanced apoptosis, repression of MADD and PIK3R2, downregulation of MEK/ERK and PI3K/AKT signaling, and repression of MCL1. Lastly, *miR-3151* methylation was significantly associated with methylation of *miR-203* and *miR-34b/c* in primary CLL samples. Therefore, this study showed that *miR-3151* is a tumor suppressive miRNA frequently hypermethylated and hence silenced in CLL. *miR-3151* silencing by DNA methylation protected CLL cells from apoptosis through over-expression of its direct targets MADD and PIK3R2, hence constitutive activation of MEK/ERK and PI3K/AKT signaling respectively, and consequently over-expression of MCL1.

## INTRODUCTION

B-cell chronic lymphocytic leukemia (CLL), the most common blood cancer in the Western countries, is characterized by accumulation of neoplastic, small B lymphocytes expressing CD5, CD19 and CD23 in bone marrow, peripheral blood and other lymphoid tissues [1]. CLL cells are protected from apoptosis by autocrine proliferation signals and survival stimuli from the microenvironment [2, 3]. It is well known that B-cell receptor (BCR) signaling is important in CLL pathophysiology, resulting in constitutive activation of downstream survival pathways including PI3K/AKT, MEK/ERK or NF-κB pathways, thereby protecting CLL cells from apoptosis, and hence have been exploited as targets for molecular therapies [4, 5].

DNA methylation refers to the addition of a methyl group (−CH3) to the carbon 5 of the cytosine ring, leading to the formation of 5-methylcytosine [6]. Many human cancers are characterized by a global DNA hypomethylation and aberrant, gene-specific DNA hypermethylation of tumor suppressor genes (TSGs) [7]. To date, numerous TSGs involved in the dysregulation of signaling pathways, including *SHP1* and *SOCS1* regulating JAK/STAT signaling, soluble *WNT* inhibitors regulating WNT signaling and *CDKN2A* and *CDKN2B* regulating the cell-cycle, have been found to be hypermethylated in multiple hematological cancers [8–10]. Of note, recent studies have identified methylation of additional TSGs including *DAPK1*, *ID4* and *SFRP1*, which are implicated in the pathogenesis or prognosis of CLL [11–13].

microRNAs (miRNAs) have been discovered as a class of single-stranded, non-protein-coding small RNAs of 19 to 25 nucleotides in length. miRNAs regulate expression of protein-coding genes by binding to specific seed region binding site on 3′ untranslated region (3′UTR) of the target mRNAs, leading to translational repression or mRNA degradation of the target genes [14]. miRNAs involved in CLL may be oncogenic or tumor suppressive. Recently, a miRNA signature comprising 18 putative oncogenic miRNAs and seven putative tumor suppressor miRNAs has been identified in CLL by comparing CLL cells with normal B cells [15]. An alternative mechanism of miRNA silencing in cancer is DNA methylation of promoter-associated CpG island. Indeed, methylation silencing of tumor suppressor miRNAs including *miR-203*, *miR-124-1*, *miR-34b/c*, and *miR-9-3* have been demonstrated in CLL, hence implicated in CLL leukemogenesis [16–19].

Recently, the updated GWAS meta-analysis, which included a total of 3,100 individuals with CLL cases and 7,667 controls, identified 12 new CLL susceptibility loci, some of which harbor apoptosis-regulating tumor suppressor genes (such as *BIM*, *NOXA*) downregulated in CLL [20]. These finding prompted us to postulate that tumor suppressive miRNAs might be present in the neighborhood of these susceptibility loci, and their expression silenced by DNA hypermethylation. Interestingly, bioinformatics analysis showed that *miR-3151*, with a promoter-associated CpG island, is localized to intron 1 of the brain and acute leukemia cytoplasmic (*BAALC*) gene at 8q22.3, one of the GWAS-identified risk loci. Herein, we studied *miR-3151* methylation in a representative cohort to define its role in CLL pathogenesis.

## RESULTS

### MSP of *miR*-3151 in normal controls and CLL cell lines

**Figure 1:**
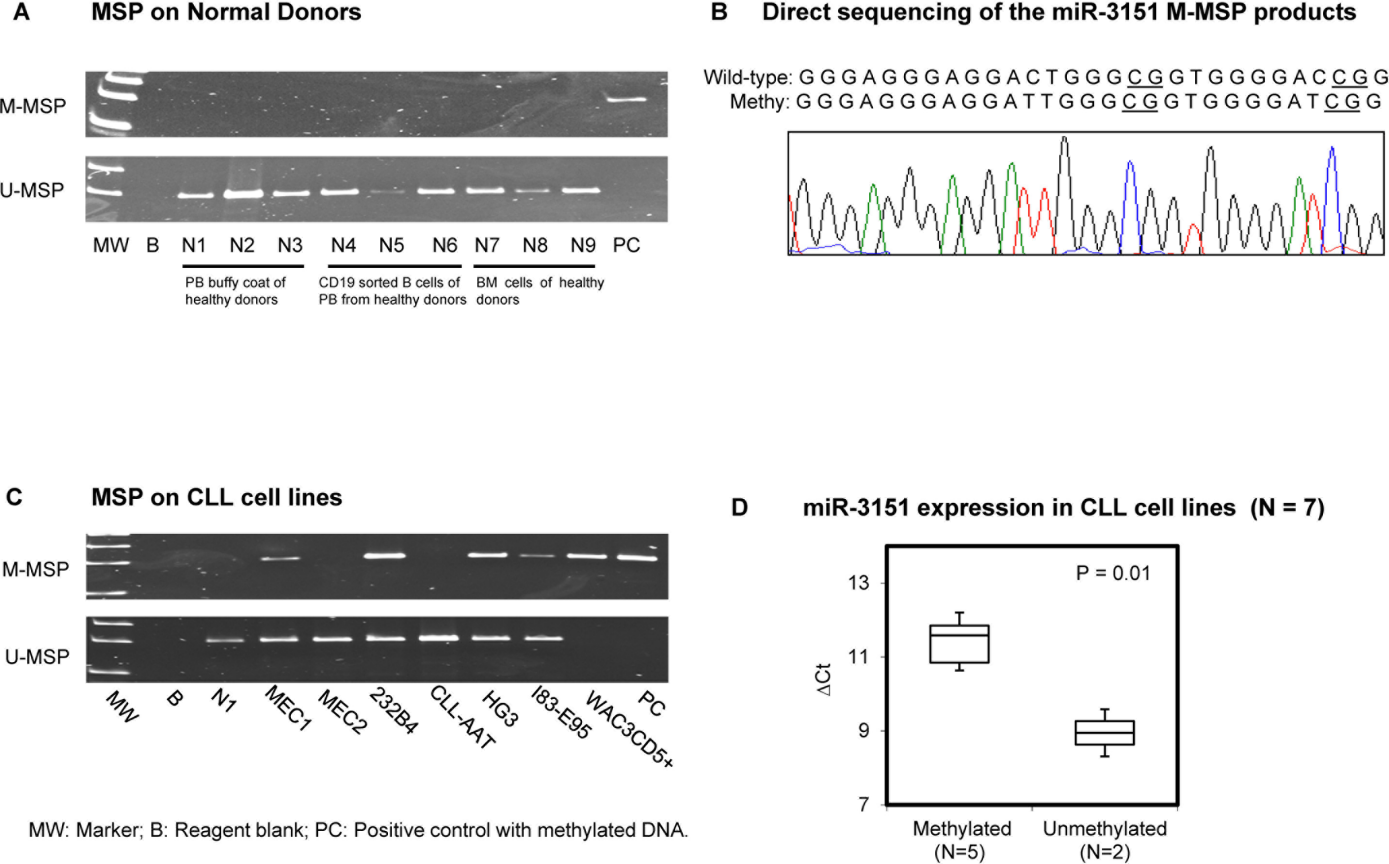
Methylation of *miR-3151* in normal donors and cell lines (**A**) M-MSP of *miR-3151* showed that the positive control (PC) was completely methylated while all 9 normal controls were completely unmethylated. In the U-MSP, the methylated control PC was totally unmethylated, but all normal controls are totally methylated. (**B**) Sequence analysis of the *miR-3151* M-MSP product from bisulfite-treated positive control DNA showed that the cytosine (C) residues of CpG dinucleotides were methylated and remained unchanged, whereas all the other C residues were unmethylated and were converted to thymidine [T], suggesting complete bisulfite conversion and specificity of MSP. (**C**) In 7 CLL cell lines, WAC3CD5+ revealed complete methylation, MEC1, 232B4, HG3 and I83-E95 were partially methylated, while MEC2 and CLL-AAT were completely unmethylated of *miR-3151*. (**D**) Stem-loop quantitative RT-PCR analysis of *miR-3151* expression in methylated (*N* = 5) versus unmethylated CLL cell lines (*N* = 2). ΔCt, Ct miR-3151-Ct RNU48. RNU48 was used as reference of *miR-3151* expression by 2^−ΔΔCT^ method.

As illustrated in the schematic diagram showing the promoter region of *miR-3151* and its host gene *BAALC* (Supplementary Figure S1A), methylation of the promoter of *miR-3151* was studied by MSP and bisulfite pyrosequencing using primers designed at the CpG island upstream to the transcription start site (TSS). None of the 9 healthy donor samples (N1 to N9) showed aberrant methylation of *miR-3151* (Figure 1A). Direct sequencing analysis of the *miR-3151* M-MSP products from a bisulfite-treated methylation positive control showed expected conversion of unmethylated cytosine to thymidine after PCR (but not methylated cytosine), indicating complete bisulfite conversion and MSP specificity (Figure 1B). Moreover, in CLL cell lines, WAC3CD5+ revealed complete methylation, MEC1, 232B4, HG3 and I83-E95 showed partial methylation, whereas MEC2 and CLL-AAT displayed complete unmethylation of *miR-3151* (Figure 1C). Quantitative bisulfite pyrosequencing validated the MSP methylation statuses (MM, MU, UU) of the CLL cell lines (Supplementary Figure S2). In addition, the mean expression of *miR-3151* in methylated CLL cell lines was significantly lower than that in unmethylated cell lines, and hence a higher ΔCt (ΔCt *miR-3151* – ΔCt *RNU48*) (*P* = 0.01) (Figure 1D).

To examine if the expression of *miR-3151* correlated to that of its host gene *BAALC* in CLL, the expression of *BAALC* was studied by qRT-PCR using Taqman assay in seven CLL cell lines. Results showed that the expression of *BAALC* in partially methylated cell lines (MEC1, 232B4, HG3 and I83-E95) was significantly lower than that of completely unmethylated CLL cell lines (MEC2 and CLL-AAT) (*P* = 0.05) (Supplementary Figure S1B), as indicated by a higher ΔCt (Ct *BAALC* - Ct *GAPDH*) in partially methylated than completely unmethylated CLL cell lines. Therefore, expression of *miR-3151* was correlated with that of its host gene *BAALC* in most CLL cell lines, except the completely methylated cell line (WAC3CD5+ cells).

### MSP of *miR-3151* in CLL primary samples at diagnosis

**Figure 2:**
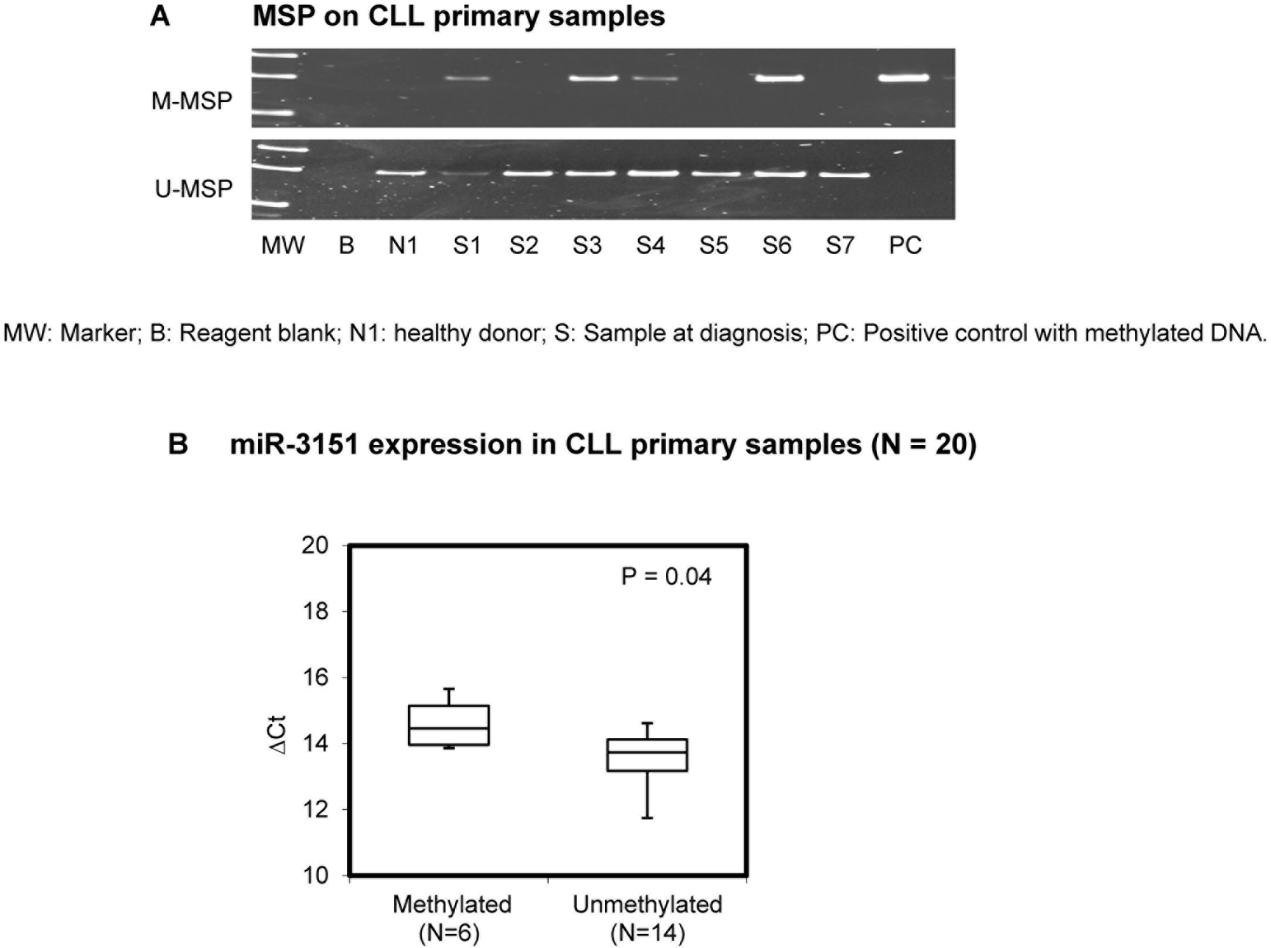
Methylation of *miR-3151* in primary samples (**A**) Promoter methylation of *miR-3151* in CLL primary samples. (**B**) Quantitative RT-PCR analysis of *miR-3151* expression between *miR-3151* methylated primary samples (*N* = 6) and unmethylated samples (*N* = 14). ΔCt, Ct *miR-3151*-Ct RNU48. RNU48 was regarded as internal control. Error bars represent standard deviation.

Methylation of *miR-3151* was detected in 30/98 (31%) of primary samples at diagnosis (Figure 2A). Importantly, in 20 samples with both DNA and RNA, methylation of *miR-3151* (*N* = 6) was associated with lower *miR-3151* expression than those without *miR-3151* methylation (*N* = 14, *P* = 0.04) (Figure 2B). There was no significant correlation between *miR-3151* methylation and the diagnostic lymphocyte count, hemoglobin level, platelet count, age, gender or high-risk karyotypes. The median OS of CLL patients with and without *miR-3151* methylation were 89 and 69 months respectively (*P* = 0.65). Among the 98 samples, concomitant methylation study of *miR-3151* with *miR-203* was performed in 50 patients, *miR-34b/c* in 78 patients, and *miR-34a* in 78 primary CLL samples respectively. The data for methylation of each of *miR-203*, *miR-34b/c* or *miR-34a* have been published previously [16, 18]. Of these, five (10%) patients showed concomitant methylation of *miR-203* and *miR-3151* (*P* = 0.002), 10 (13%) patients concomitant methylation of *miR-34b/c* and *miR-3151* (*P* = 0.001), and two (2.6%) patients concomitant methylation of *miR-34a* and *miR-3151* (*P* = 0.07).

### 5-AzadC treatment of WAC3CD5+ and MEC2 cells

**Figure 3:**
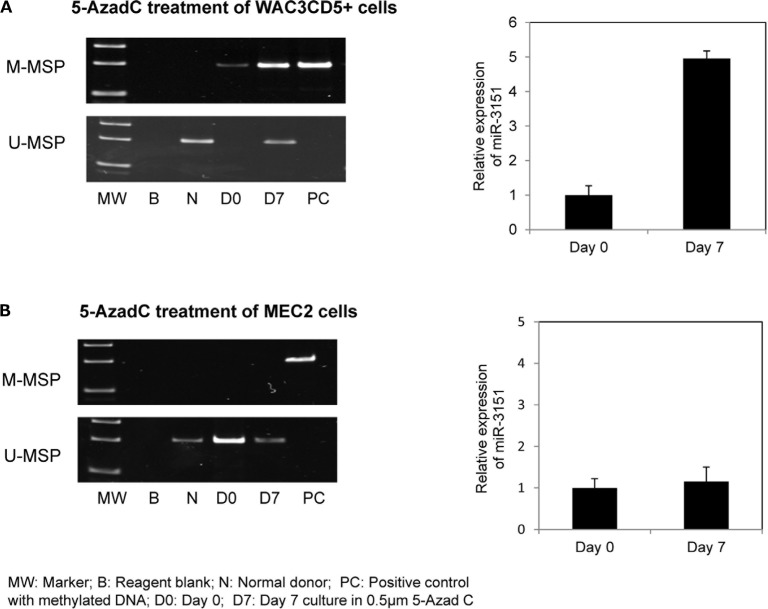
Effect of 5-Aza-2′-deoxycytidine (5-AzadC) treatment on WAC3CD5+ and MEC2 cells U-/M-MSP of *miR-3151* promoter methylation status and stem-loop quantitative RT-PCR analysis of mature *miR-3151* expression in (**A**) WAC3CD5+ cells and (**B**) MEC2 cells before and after treatment with 0.5 μM 5-AzadC for 7 days. ΔCt, Ct*miR-3151*-Ct RNU48. RNU48 was used as reference for data analysis of *miR-3151* expression by 2^−ΔΔCT^ method.

To test if promoter methylation led to downregulated expression of *miR-3151*, both CLL cell lines completely methylated (WAC3CD5+) and completely unmethylated (MEC2) for *miR-*3151 were independently treated with a hypomethylating agent. Treatment with 5-AzadC in WAC3CD5+ cells led to promoter demethylation as evidenced by the emergence of U-MSP signal, and hence, with a corresponding increase of *miR-3151* and *BAALC* expression on Day 7 (Figure 3A, Supplementary Figure S1C and Figure S2C). By contrast, treatment with 5-AzadC in MEC2 cells did not render a change in the methylation status and expression of *miR-3151* (Figure 3B).

### Effect of *miR-3151* over-expression in WAC3CD5+ cells

**Figure 4:**
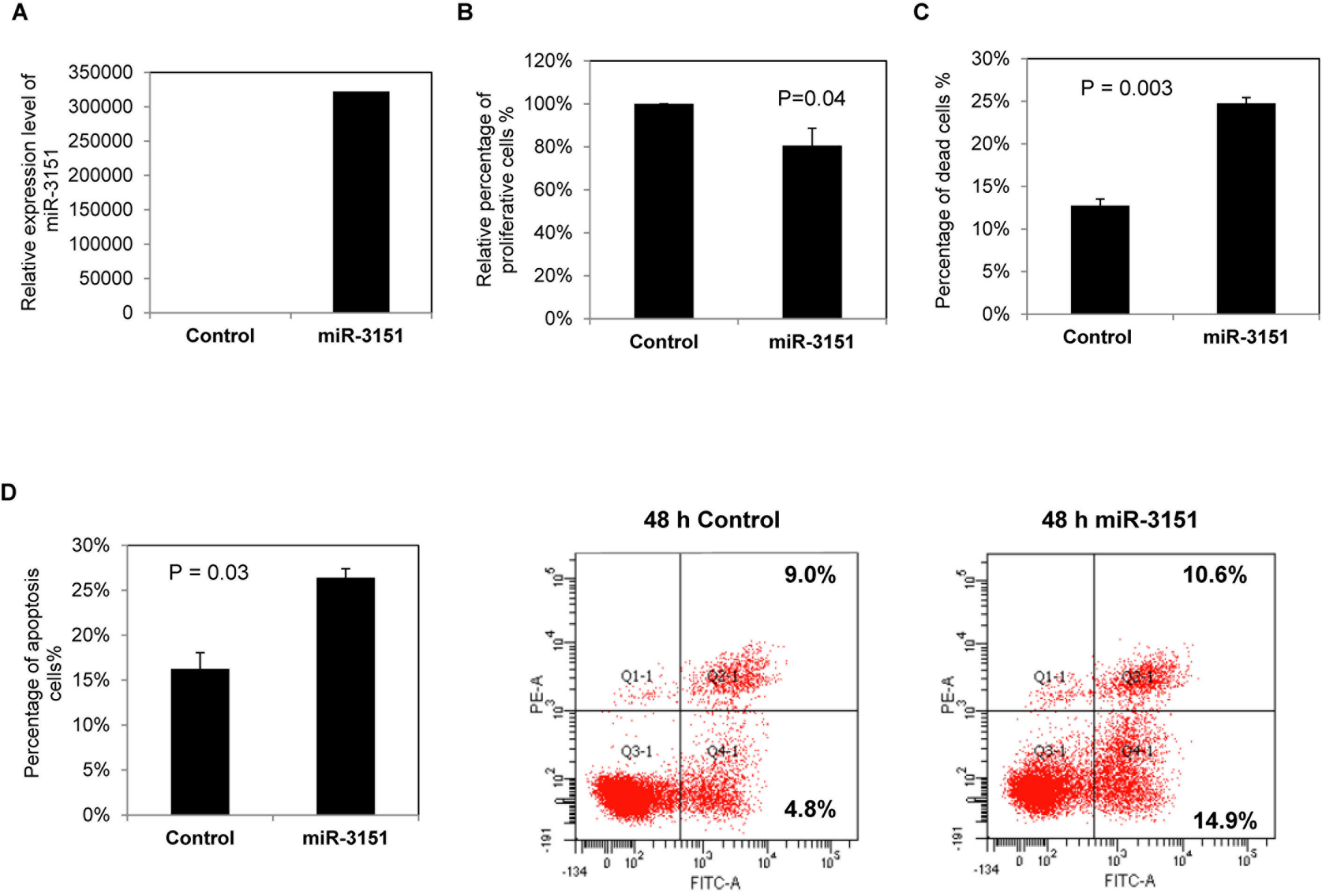
Over-expression of *miR-3151* in CLL cells WAC3CD5+ cells, completely methylated for *miR-3151*, were transfected with *miR-3151* mimic or scrambled oligonucleotides control. Cells were harvested for functional assays 48 hours after transfection. (**A**) Stem-loop quantitative RT-PCR analysis of *miR-3151* expression at 48 hours after transfection. ΔCt, Ct *miR-3151*-Ct RNU48. RNU48 was chosen as reference using the 2^−ΔΔCT^ method. (**B**) Relative cell proliferation of CLL cells upon over-expression of *miR-3151* was measured by MTT assay. Column, mean percentage of cell proliferation from three experiments conducted in triplicate. (**C**) Percentage of dead cells was measured by Trypan blue exclusion assay. Column, mean percentage of cell death from three experiments with triplicate in each. (**D**) The percentage of apoptotic CLL cells after *miR-3151* transfection was assessed by flow cytometry using FITC Annexin V-PI staining. Left panel: represented average percentage of apoptotic cells from three independent experiments. Right panel: representative results. Error bars represent standard deviation.

As *miR-3*151 is frequently methylated and under-expressed in CLL cell lines and primary samples, we postulated that it might act as a tumor suppressive miRNA, and hence investigated tumor suppressor function of *miR-3151* in CLL cells *in vitro*. By TaqMan stem-loop quantitative RT-PCR, over-expression of *miR-3151* was shown to be induced in WAC3CD5+ cells 48 hours after transfection (Figure 4A). Overexpression of *miR-3151* led to a 20% reduction of cell proliferation as measured by MTT assay (*P* = 0.04, Figure 4B), an addition of 12% dead cells by Trypan blue exclusion assay (*P* = 0.003, Figure 4C) and an addition of 10% apoptosis cells as shown by Annexin V and PI staining (*P* = 0.03, Figure 4D), suggesting a tumor suppressive role of *miR-3151* in CLL.

### Identification of PIK3R2 and MADD as direct targets of *miR-3151*

To further understand the mechanism by which *miR-3151* regulates cell proliferation and apoptosis in CLL cells, potential direct targets of *miR-3151* were screened using four computational prediction softwares including TargetScan Version 6.2, DIANA-microT v3.0, miRDB and miRanda. Two putative targets, *MADD* and *PIK3R2*, with known oncogenic properties were concomitantly predicted by all these four miRNA target prediction algorithms, and hence were further studied.

**Figure 5:**
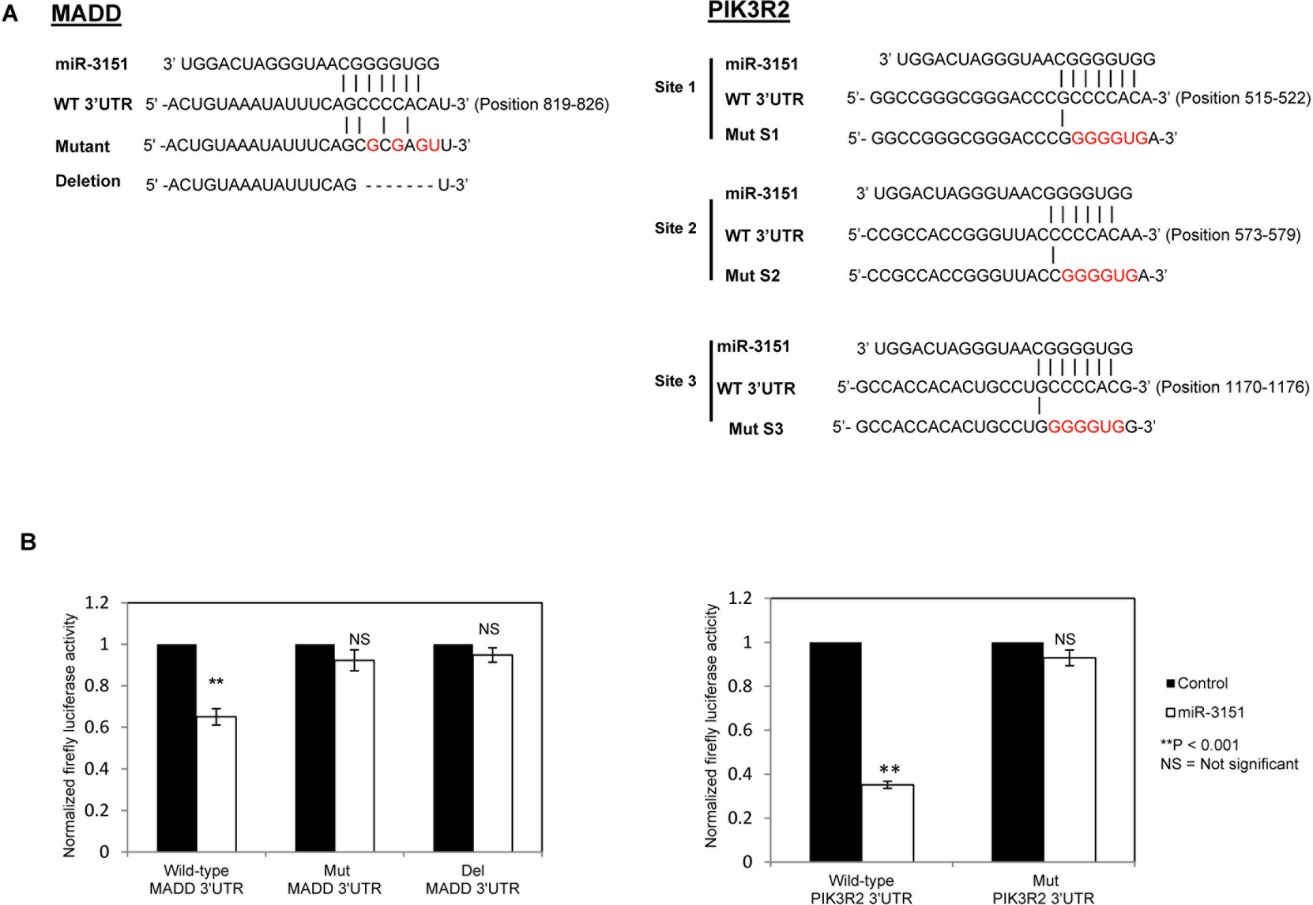
Identification of direct targets MADD and PIK3R2 for *miR-3151* (**A**) Sequence complementarity of potential *miR-3151* binding sites in *MADD*-3′UTR and *PIK3R2*-3′UTR. 3′UTR of MADD includes one putative *miR-3151* binding site while 3′UTR of *PIK3R2* is composed of three putative *miR-3151* binding sites (S1–S3). Corresponding mutant 3′UTR are shown below. The mutated nucleotides in the 3′UTR of MADD or PIK3R2 are indicated in red. Dash line, deletion region in 3′UTR. (**B**) Luciferase constructs carrying wild-type or mutant 3′UTR of target gene (*MADD* or *PIK3R2*) was co-transfected with *miR-3151* as compared with scrambled oligonucleotides control in HeLa cells for 48 hours. Mut *PIK3R2*-3′UTR contained a three mutated sites, namely Mut S1, Mut S2 and Mut S3, simultaneously. Del *MADD*-3′UTR was deleted the binding site sequence (5′-CCCCACA-3′). Firefly luciferase activity was normalized to the Renilla lucifrease activity. Column, mean of normalized luciferase activity in triplicates after three independent transfections. Error bar represents standard deviation.

To confirm *MADD* and *PIK3R2* to be direct targets of *miR-3151*, wild-type and mutant 3′UTR of *MADD* and *PIK3R2* luciferase reporter constructs were generated (Figure 5A). For each of *MADD* and *PIK3R2*, the wild-type or mutant luciferase construct was co-transfected with *miR-3151* mimic into HeLa cells for 48 hours. As shown in Figure 5B, as compared with co-transfection of scrambled oligonucleotides control, a 35% reduction of luciferase activity was observed in *miR-3151* co-transfected with wild-type *MADD*-3′UTR. This suppression was abolished when *miR-3151* was co-transfected with Mut *MADD*-3′UTR (with 4 point mutations in seed region binding site) or Del *MADD*-3′UTR (with *miR-3151* binding site deleted). Similarly, as compared with the scrambled oligonucleotides control, over-expression of *miR-3151* with wild-type *PIK3R2-*3′UTR led to a 65% reduction of luciferase activity. By contrast, the luciferase activity was not suppressed by *miR-3151* when 6 point mutations were introduced in each of the three *miR-3151* binding sites on the 3′UTR of *PIK3R2* (Mut *PIK3R2*-3′UTR). These findings confirmed that *MADD* and *PIK3R2* are both direct targets of *miR-3151*. Moreover, in CLL cell lines, whether methylation and hence silencing of *miR-3151* correlates with increased expression of *MADD* and *PIK3R2* was studied by qRT-PCR. Results showed that a trend of methylation was associated with higher expression of the target genes, as indicated by the lower ΔCt (Ct *MADD/PIK3R2* - Ct *GAPDH*) in methylated cell lines (MM or MU), as compared with those in unmethylated cell lines (UU) (Supplementary Figure S3), confirming the role of methylation of *miR-3151* in the regulation of *MADD* and *PIK3R2* in CLL cells.

### *miR-3151* overexpression suppresses CLL cell proliferation by directly targeting MADD and PIK3R2 implicated in MEK/ERK and PI3K/AKT signaling

**Figure 6:**
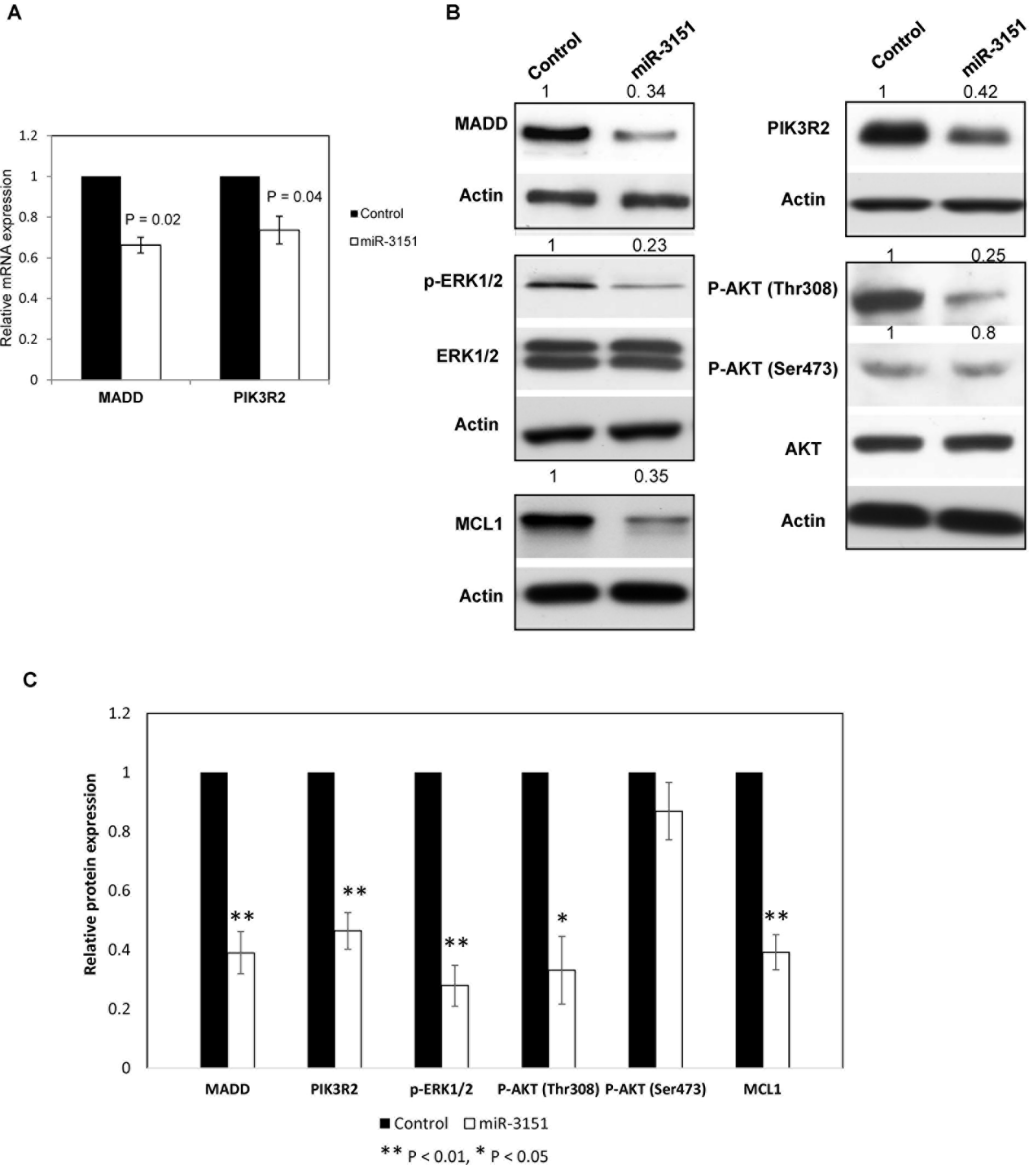
*miR-3151* regulates the MEK/ERK and PI3K/AKT signaling by repressing MADD and PIK3R2 respectively (**A**) *MADD* and *PIK3R2* mRNA expression was downregulated upon *miR-3151* transfection in WAC3CD5+ cells as compared to scrambled oligonucleotides control. ΔCt, Ct *MADD/PIK3R2*-Ct GAPDH. *GAPDH* was used as reference for data analysis of *MADD/PIK3R2* expression by 2^−ΔΔCT^ method. Error bars represent standard deviation. (**B**) Western analysis showed a decrease of MADD and PIK3R2 protein by introduction of *miR-3151* mimic in WAC3CD5+ cells, followed by the reduced p-ERK1/2, P-AKT (Thr308) and MCL1 protein, instead of P-AKT (Ser473). Total levels of ERK1/2 and AKT were not affected. For MADD, PIK3R2 and MCL1, Actin was regarded as the endogenous normalizer and densitometric analysis of normalized protein levels are indicated above the western blot. (**C**) Two independent transfection were performed. Columns represent the average of quantitative densitometry of the relative protein expression (including MADD, PIK3R2, P-ERK1/2, P-AKT (Thr308), P-AKT (Ser473) and MCL1) from two independent experiments. Error bars represent standard deviation.

miRNAs may affect mRNA stability or translation of target protein-coding genes [21]. To address the biological significance of *miR-3151* methylation in CLL cells, we evaluated the mRNA and protein expression of its target genes by qRT-PCR and Western blot respectively after over-expression of *miR-3151* in WAC3CD5+ cells with complete *miR-3151* methylation. Upon *miR-3151* over-expression, *MADD* and *PIK3R2* mRNA levels were significantly downregulated by 34% (*P* = 0.02) and 28% (*P* = 0.04) respectively (Figure 6A).

Moreover, at the protein level, restoration of *miR-3151* in completely methylated cells resulted in 66% downregulation of MADD protein, in association with 77% repression of the downstream phospho-ERK1/2 protein, indicating that *miR-3151* may function as a tumor suppressor by inhibiting MADD/MEK/ERK signaling (Figure 6B). Furthermore, *miR-3151* over-expression also led to the 58% downregulation of PIK3R2 protein, which was associated with a 75% reduction of the downstream phospho-AKT (Thr308) protein but not phospho-AKT (Ser473), showing that *miR-3151* methylation/silencing accounted for constitutive PIK3R2/PI3K/AKT activation in CLL (Figure 6B). Finally, MCL1 is an important anti-apoptotic protein upregulated by ERK or AKT signaling in many cancers including CLL. As shown in Figure 6B, MCL1 protein expression was repressed by 65% upon *miR-3151* over-expression, thereby testifying the tumor suppressor function of *miR-3151* in CLL. The average of quantitative densitometry of the relative protein expression (including MADD, PIK3R2, P-ERK1/2, P-AKT (Thr308), P-AKT (Ser473) and MCL1) from two independent experiments were plotted as shown in Figure 6C.

To further confirm the role of *PIK3R2* and *MADD* in *miR-3151*-mediated tumor suppression in CLL cells, knockdown of each of *PIK3R2* and *MADD* was performed in WAC3CD5+ cells with complete *miR-3151* methylation. Upon successful knockdown of *PIK3R2* as shown by reduced levels of both mRNA and protein (Supplementary Figure S4A-B), a 30% reduction of cell proliferation by MTT assay (*P* = 0.04) (Supplementary Figure S4C) and a 13% increase of dead cells measured by Trypan blue exclusion assay (*P* < 0.01) were demonstrated (Supplementary Figure S4D), as compared with control siRNA. Similarly, knockdown of *MADD* in WAC3CD5+ cells (Supplementary Figure S5A-B) showed 20% decreased cellular proliferation by MTT assay (*P* = 0.01) (Supplementary Figure S5C) and 13% increased cell death by Trypan blue exclusion assay (*P* < 0.01) (Supplementary Figure S5D). Collectively, these data confirmed that the tumor suppressor function of *miR-3151* was mediated via downregulation of *PIK3R2* and *MADD* (Figure 6).

## DISCUSSION

A few observations were made in this study.

Firstly, we demonstrated the tumor-specific methylation of *miR-3151* in CLL as evidenced by frequent methylation in CLL cell lines and primary samples but not normal controls, including CD19-sorted normal B-cells. By contrast, *miR-9-2* and *miR-373* are methylated in both tumor cells and the corresponding normal counterparts, indicating a tissue-specific and not tumor-specific methylation pattern [17, 22].

Secondly, DNA methylation of *miR-3151* was associated with reversible gene silencing. This was supported by, first, an inverse correlation between methylation status and expression of *miR-3151* in cell lines and primary CLL cells. Moreover, hypomethylation treatment of WAC3CD5+ cells with 5-AzadC, which was completely methylated of *miR-3151*, led to demethylation and re-expression of *miR-3151*.

Thirdly, we showed that *miR-3151* is a tumor suppressor miRNA in CLL, in contrast to recent reports that *miR-3151* is oncogenic in cytogenetically normal acute myeloid leukemia (AML) [23, 24], in which *miR-3151* functioned as the oncogenic driver targeting *TP53* [25]. By contrast, herein, *miR-3151* is tumor suppressive in CLL as evidenced by decreased cell proliferation and enhanced apoptosis upon over-expression. Similarly, *miR-9* has been shown to be tumor suppressive in ovarian cancer and CLL via inhibition of NF-κB pathway but oncogenic in breast cancer via activation of β-catenin signaling [17, 26, 27]. Therefore, a miRNA can be oncogenic in one but tumor suppressive in another cancer type.

Fourthly, *in vitro* activation of BCR signaling by anti-IgM BCR antibodies induced activation of both MEK/ERK and PI3K/AKT signaling in CLL, thereby protecting CLL cells from apoptosis [4, 28]. Indeed, by luciferase assays, MADD and PIK3R2 have been validated as direct novel targets of *miR-3151*. MADD, a splice variant of IG20 (insulinoma-glucagonoma 20) overexpressed in cancer cells [29], is crucial for RAS/MEK/ERK activation by facilitating recruitment of Grb2 to the receptor-mediated signaling complex [30]. On the other hand, PIK3R2 is a subunit of a protein complex for activation of PI3K signaling, which comprises p85 (p85α and p85β [alias PIK3R2]) and a p110 catalytic subunit (α, β, γ) [31]. Indeed, the oncogenic role of PIK3R2 has been demonstrated in breast and colon cancer, in which over-expression of *PIK3R2* with concomitant AKT activation correlated with tumor progression *in vivo* [32].

Herein, over-expression of *miR-3151* promoted apoptosis of CLL cells via repression of MADD and p-ERK1/2, and hence inhibition of MADD/RAS/ERK signaling. On the other hand, functional activation of AKT is dependent on the phosphorylation of two residues, Ser473 in the hydrophobic motif or Thr308 in the activation loop [33]. Recently, Thr308 phosphorylation has been found a better biomarker than Ser473 phosphorylation for AKT activation [34]. Similarly, our data showed that the over-expression of *miR-3151* enhanced apoptosis of CLL cells through suppression of PI3K/AKT pathway by repressing PIK3R2, which was associated with profound downregulation of Thr308 but not Ser473 phosphorylation. Therefore, activation of PI3K signaling associated with *miR-3151*-methylation in CLL is mediated by Thr308 but not Ser473 phosphorylation.

In cancer, both ERK and AKT signaling confer resistance to apoptosis via upregulation of a panel of anti-apoptotic proteins including MCL1, X-linked inhibitor of apoptosis protein (XIAP) and BCL-xL. MCL1 has been shown to inhibit apoptosis in CLL [35–37]. Indeed, restoration of *miR-3151* mimic in CLL cells resulted in repression of MCL1 protein. Taken together, our data suggested that *miR-3151* methylation led to constitutive ERK and AKT activation, and hence over-expression of MCL1, thereby protecting CLL cells from apoptosis.

Last but not least, methylation of *miR-3151* (chr. 8q22.3) was associated with that of *miR-203* (chr 14q32) and *miR-34b/c* (chr 11q23), which are localized to different chromosomal regions. *miR-34b/c* and *miR-203* have also been shown to target CREB independently, and hence methylation of these miRNAs may render upregulation of multiple oncogenes including BCL2 and NF-κB1 [38, 39]. Therefore, these four tumor suppressor miRNAs undergoing methylation may collaborate to confer proliferation advantages to CLL cells.

**Figure 7:**
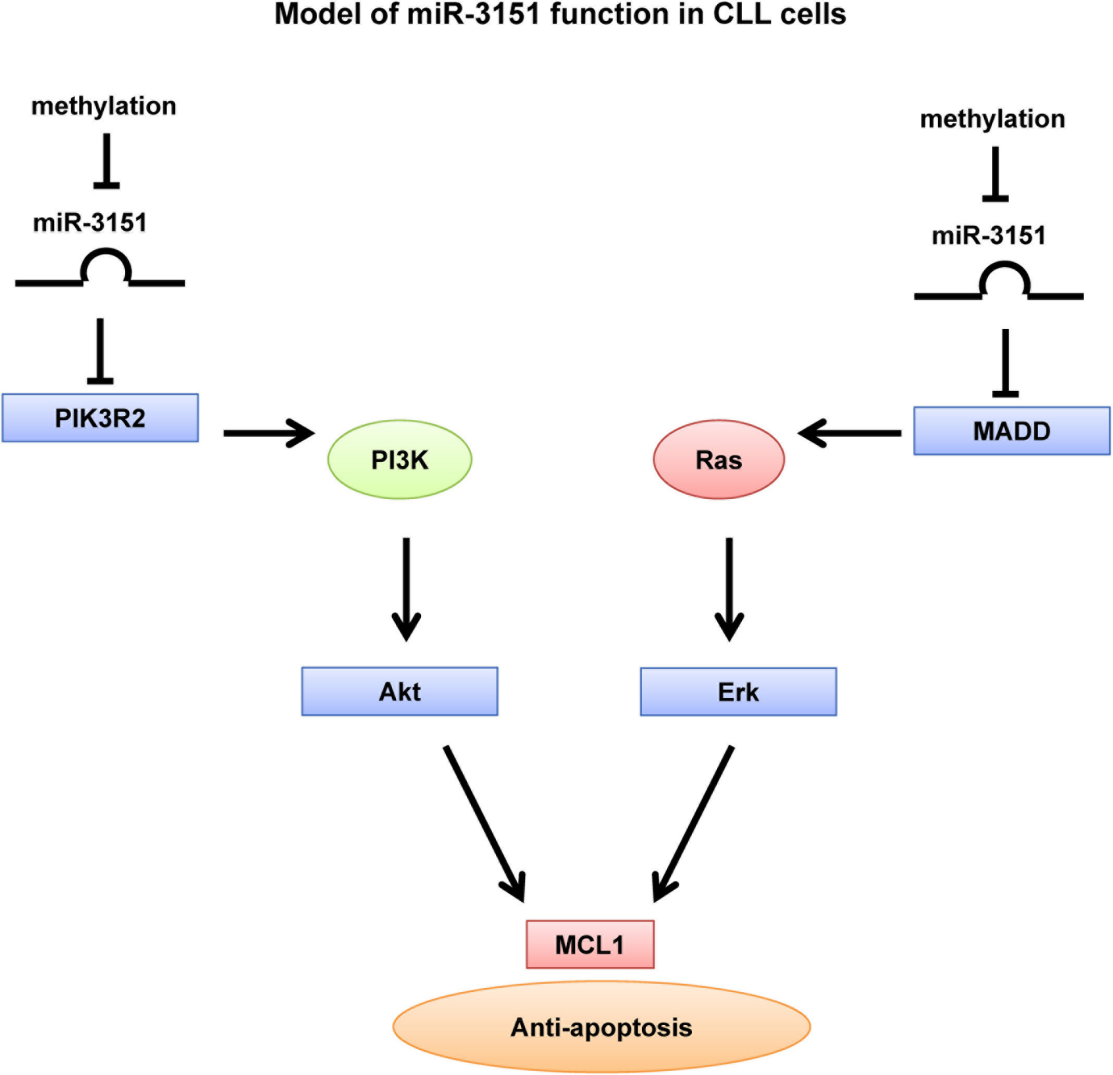
Model of *miR-3151* function in CLL cells Over-expression of *miR-3151* represses PIK3R2 and MADD, which further downregulate the PI3K/AKT and MEK/ERK signaling respectively. Thus, *miR-3151* methylation protected CLL cells from apoptosis through over-expression of direct targets PIK3R2 and MADD, leading to constitutive activation of PI3K/AKT and MEK/ERK signaling respectively, with consequent upregulation of MCL1.

In conclusion, our results showed that *miR-3151* was a tumor suppressor miRNA frequently methylated and hence silenced in CLL. Methylation of *miR-3151* in CLL cells conferred resistance to apoptosis through over-expression of its direct targets MADD and PIK3R2, and hence constitutive activation of RAS/MEK/ERK and PI3K/AKT, and consequently over-expression of MCL1 (Figure 7).

## MATERIALS AND METHODS

### Patient samples

Bone marrow samples were collected from 98 Chinese CLL patients diagnosed according to the standard morphologic and immunophenotyping criteria as previously [17, 40]. Patient demographics were listed in Table 1. The median overall survival (OS) of this cohort was 89 months. The median OS of those with advanced Rai stage and limited Rai stage were 57 and 111 months respectively (*P* = 0.006). Moreover, the median OS for those with or without high/intermediate-risk karyotype were 43 months and 111 months respectively (*P* = 0.04). Of these, the *miR-203* (*N* = 50), *miR-34b/c* (*N* = 78) and *miR-34a* (*N* = 78) methylation results have been reported previously [16, 18]. This study was approved by the Institutional Review Board of Queen Mary Hospital and samples were collected with written informed consent and in accordance with the Declaration of Helsinki.

**Table 1:** The CLL patients demographics (*N = 98*)

Characteristic	Value
Gender (M/F)	67 (68.4%)/31 (31.6%)
Median age (range)—yr	67 (37–91)
Rai stage (≤ II/> II)*	56 (62.2%)/34 (37.8%)
Median lymphocyte count (range)	16 × 10^9^/L (10–540 × 10^9^/L)
**High-risk cytogenetics**^‡^	
*del (17p)*	3 (4.8%)
*del (11q)*	2 (3.2%)
*trisomy 12*	11 (17.5%)
**Low-risk cytogenetics**^‡^	
*del (13)*	19 (30.2%)
*normal karyotype*	21 (33.3%)
*other karyotype abnormalities*	7 (11.0%)

* from 90 patients with clinical data;

‡ from 63 patients with cytogenetic data.

### Cell lines and culture

The human CLL cell lines MEC1 and CLL-AAT were purchased from Deutsche Sammlung von Mikroorganismen und Zellkulturen Deutsche GmbH (DSMZ) (Braunschweig, Germany) and American Type Culture Collection (ATCC) (Manassas, USA) respectively. MEC2 [41], I83-E95 [42] and WAC3CD5+ [42] were kind gifts from Dr John C. Byrd, Department of Medicine, Ohio State University. HG3 and 232B4 were previously established by Prof. Anders Rosén, Department of Clinical & Experimental Medicine, Linköping University [43, 44]. Cell lines were cultured in 90% RPMI 1640 + 10% FBS, supplemented with 50 U/ml penicillin and 50 μg/ml streptomycin (Invitrogen, Carlsbad, CA, USA) in a humidified atmosphere of 5% CO_2_ at 37°C.

### Methylation-specific polymerase chain reaction (MSP)

DNA was extracted from 98 primary samples, 7 cell lines and 9 normal controls (peripheral blood buffy coats from healthy donors, *N* = 3; CD19 sorted peripheral blood B cells from healthy donors, *N* = 3; and bone marrow buffy coats from healthy donors, *N* = 3) using the QIAamp DNA Blood Mini Kit (QIAGEN, Germany) and then were treated with bisulfite to convert unmethylated cytosine to uracil (but unaffecting methylated cytosine) by the EpiTect Bisulfite Kit kit (QIAGEN, Hilden, Germany). For each bisulfite-treated sample, two sets of primers were used to amplify the methylated DNA (methylated-MSP, M-MSP) and unmethylated DNA (unmethylated-MSP, U-MSP) respectively. Primers and conditions for M-MSP and U-MSP of *miR-3151* were listed in Table 2.

**Table 2:** *miR-3151* MSP Primer sequences and condition

*miR-3151*	Forward primer (5′ to 3′)	Reverse primer (5′ to 3′)	Tm/cycles/MgCl_2_
**U-MSP**	GTTTTTGTTTTTGGGTTGTTAT	AAAACTCCTACAATACCCA	56°C /35 × /2 mM
**M-MSP**	GTTTTTGTTTTCGGGTCGTTAC	CGAAACTCCTACGATACCCG	55°C/35 × /1.5 mM

Abbreviations: M-MSP, MSP for the methylated allele; U-MSP, MSP for the unmethylated allele; Tm, annealing temperature.

### Quantitative bisulfite pyrosequencing

Primers for pyrosequencing were used for the amplification of *miR-3151* promoter region, which was overlapped with the amplicon of MSP. Primers were designed by PSQ Assay Design software (Biotage). Bisulfite-treated DNA was amplified by PCR with a forward primer (5′-ATGGATAGATGGGTTAGAGATTG-3′) and a biotinylated reverse primer (5′-AATAAACCAAATAAATTCTATCTCC-3′) in the following condition: 2 mM/59°C/50X. A stretch of DNA with 6 adjacent CpG dinucleotides was pyrosequenced using sequencing primer: 5′- GAGAGTTTAGGGAGTTTTAGTTTT-3′. Bisulfite-treated DNA (2μl) was used as template in a PCR reaction with final volume of 50μl. The PCR products were purified and loaded onto 6% non-denaturing polyacrylamide gels with a mass ladder (Fermentas) and stained with ethidium bromide staining for sizing and quantification. Biotin-labeled PCR fragments (5 pmoles) of each sample were subjected to pyrosequencing using the PSQ96MA System (Biotage) and analyzed using PyroQ-CpG 1.0.9, according to manufacturer's instructions.

### 5-Aza-2′-deoxycytidine (5-AzadC) treatment

Each of WAC3CD5+ and MEC2 cells at log-phase was cultured in six-well plates at a density of 10^6^ cells/ml, with 0.5 μM of 5-AzadC (Sigma-Aldrich, St. Louis, MO, USA) for 7 days. Fresh 5-AzadC was replaced every 24 hours. Cells of 5-AzadC treatment on day 0 and day 7 were harvested.

### Quantification of *miR-3151*, *BAALC*, *MADD* and *PIK3R2*

Total RNA was extracted by the mirVana miRNA Isolation Kit (Ambion, Austin, TX, USA). *miR-3151* was quantified by the TaqMan MicroRNA RT Kit and TaqMan MicroRNA Assay Kit (ABI, Foster City, CA, USA) with RNU48 as reference. *BAALC* was quantified by *BAALC* Taqman Gene Expression Assay (Cat. 4351372) (ABI, Foster City, CA, USA), according to the manufacturer's instructions. For *MADD* and *PIK3R2*, total RNA was reverse transcribed by the QuantiTect Reverse Transcription Kit (QIAGEN, Valencia, CA), and quantified using SYBR Green Master Mix (ABI, Foster City, CA, USA), following the manufacturer's instructions. *GAPDH* was used as endogenous control for data analysis of *BAALC*, *MADD* and *PIK3R2* [45]. The 2^−ΔΔCT^ method was used to detect the expression of *miR-3151* and target genes (*MADD* and *PIK3R2*) before and after over-expression of *miR-3151* in WAC3CD5+ cells; and the expression of *miR-3151* before and after 5-AzadC treatment [45]. For comparing the *miR-3151* and target genes (*MADD* and *PIK3R2*) expression between methylated and unmethylated CLL cell lines or primary samples, ΔCT method is used. Primers for detecting the mRNA expression of *MADD* and *PIK3R2* were summarized in Supplementary Table S1.

### Transfection of *miR-3151*

Either precursor *miR-3151* mimic (final concentration 100 nM) (Ambion, Austin, TX, USA) or scrambled oligonucleotides control (negative control) was transfected into 1 × 10^6^ WAC3CD5+ cells at log phase using X-tremeGENE siRNA Transfection Reagent (Roche, Basel, Switzerland), according to the manufacturers' instructions.

### Silencing of *PIK3R2* and *MADD* by RNA interference

RNA interference by small interfering RNA (siRNA) was used to knockdown the expression of *PIK3R2* and *MADD* in WAC3CD5+ cells. For knockdown of *PIK3R2*, cells were plated at a density of 1 × 10^6^/ml in a six-well plate and transfected with 120 nM PIK3R2 siRNA (top strand: 5′-GCGGGAACAAUAAGCUGAUTT-3′) or control siRNA (GenePharma Biotechnology, Shanghai, China) using Lipofectamine 2000 (Invitrogen) according to the manufacturer's instructions. Cells were cultured and analyzed at 72 hours after transfection. For knockdown of *MADD*, cells were transfected with 100nM MADD siRNA (Santa Cruz Biotechnology, sc-75726) or control siRNA-A (Santa Cruz Biotechnology, sc-37007) and analyzed at 48 hours after transfection. The efficiency of siRNA was determined by qRT-PCR and Western blot.

### Cell proliferation and viability assays

MTT assay was used to determine the cellular proliferation by colorimetric quantification of purple formazan formed from the reduction of yellow tetrazolium MTT (3-(4, 5-dimethylthiazolyl-2)-2, 5-diphenyltetrazolium bromide) by proliferative viable cells [46]. Cells were seeded in a 96-well microtitre plate at 2× 10^5^ cells/well in 100 μl of medium. Forty-eight hours after transfection, each well was added 10 μl of 5 mg/ml MTT reagent (Sigma-Aldrich) and incubated for 5 hours, followed by adding 100 μl dimethyl sulfoxide (DMSO). Then the absorbance at 550 nm with reference to 650 nm was measured. Cell viability was analyzed by Trypan blue dye exclusion assay under microscope and five random microscopic fields were counted for each sample. Dead cells (%) = (total number of dead cells per microscopic field / total number of cells per microscopic field) × 100. MTT assay and Trypan blue dye exclusion assay were performed in triplicate in each of the three independent transfections.

### Cell apoptosis assay

Cellular apoptosis was verified by flow cytometry using FITC Annexin V Apoptosis Detection Kit I (BD-Pharmingen) as described before [17, 47]. The presence of cells with FITC Annexin V positive, propidium iodide (PI) negative (early apoptosis) or FITC Annexin V positive, PI positive cells (late apoptosis) suggests apoptosis. FACS analyses were performed by a flow cytometer (FACSCanto II) equipped with FACSDiva V6.1.2 software (BD Biosciences). Data were derived from three independent transfections.

### Plasmid constructs

The full-length 3′UTR of *MADD* includes one putative *miR-3151* binding site (819-826 bp of 3′UTR) while the 3′UTR of *PIK3R2* includes three putative *miR-3151* binding sites (Site 1, 515–522 bp; Site 2, 573–579 bp; Site 3, 1170-1176 bp of 3′UTR). The 3′UTR segments of *MADD* (~220 bp) and *PIK3R2* (~750 bp) containing putative *miR-3151* binding sites were respectively amplified and cloned into the NheI and SalI sites of a dual firefly/renilla luciferase reporter vector, pmirGLO (Promega, Madison, WI, USA). Two different types of *MADD*-3′UTR mutants were constructed using the QuikChange Lightning Site-Directed Mutagenesis Kit (Stratagene, La Jolla, CA, USA), one with 4 point mutations introduced in the seed region binding site (Mut *MADD*-3′UTR) while the other with the entire seed region binding site (5′-CCCCACA-3′) deleted (Del *MADD*-3′UTR). For *PIK3R2*-3′UTR mutant (Mut *PIK3R2*-3′UTR), six point mutations were introduced to each of the three binding sites by mutagenesis using QuikChange Lightning Multi Site-Directed Mutagenesis Kit (Stratagene, La Jolla, CA, USA). The primers for wild-type and mutant *MADD*/*PIK3R2*-3′UTR constructs above were summarized in Supplementary Table S1. Wild-type and mutant inserts were confirmed by sequencing shown in Supplementary Figure S6.

### Luciferase reporter assay

Luciferase reporter vector pmirGLO (Wild-type or mutant *MADD*/*PIK3R2*-3′UTR, 1 μg) was co-transfected with either precursor *miR-3151* mimic (final concentration 50 nM) or scrambled oligonucleotides control (final concentration 50 nM) into HeLa cells (kind gift from Dr Zou, Department of Medicine, The University of Hong Kong) using Lipofectamine 2000 (Invitrogen). After 48 hours transfection, the luminescent signal was generated and analyzed by Dual-Luciferase Reporter Assay System (Promega), according to the manufacturers' instructions [39]. Firefly luciferase activity was normalized to the Renilla luciferase activity. Data represents the mean normalized luciferase activity derived from three independent transfections with triplicate in each.

### Western Blotting

Western Blots were performed with 20 μg protein from each sample. After 48 hours transfection, WAC3CD5+ cells were harvested and lysed in RIPA buffer (50 mM Tris-HCl, pH 7.4, 150 mM NaCl, 0.2% SDS, 1% Triton X-100, 2 mM EDTA). Protein lysates were separated on 10% SDS-PAGE and blotted onto a 0.2 μm nitrocellulose membrane (Bio-Rad, Hercules, CA). The primary antibodies used were for anti-MADD (1:2000; Abcam), -PIK3R2 (1:1000; Abcam), -phospho-p44/42 MAPK (ERK1/2) (Thr202/Tyr204) (1:1000; Cell Signaling), -p44/42 MAPK (ERK1/2) (1:1000; Cell Signaling), -phospho-Akt (Thr308) (1:2000; Cell Signaling), -phospho-AKT (Ser473) (1:2000; Cell Signaling), -AKT (1:1000; Cell Signaling) and anti-actin (1:5000; Sigma-Aldrich, USA) at 4°C overnight. Then membranes were washed and incubated with anti-rabbit or anti-mouse horseradish peroxidase conjugate secondary antibody at room temperature for 1 hour. ECL plus Western blotting detection reagent was used for the detection of protein signals with X-ray film (Amersham Biosciences, Buckinghamshire, UK). Protein bands were quantified using densitometry as measured by Quantity One 4.6.2 software (Bio-Rad); hence relative protein expression was expressed in comparison to corresponding control.

### Statistical analysis

In 98 primary CLL samples, the correlation between *miR-3151* methylation with continuous (mean age, mean lymphocyte counts, diagnostic hemoglobin or platelet counts) and categorical variables (gender, Rai stage or high-risk karyotypes) were analyzed by student's t-test and chi-square test (or Fisher's exact test) respectively. OS is measured from the date of diagnosis to the date of last follow-up or death. Survival was plotted by the Kaplan–Meier method and compared by the log-rank test. OS of CLL patients with limited Rai stage (stage 0/I/II) was compared with those with advanced Rai stage (stage III/IV). Moreover, OS of CLL patients with high-risk karyotypes [del(17p), del(11q) or trisomy 12] was compared to those with standard-risk karyotypes [del(13q), normal karyotype or other karyotypic changes]. Association between methylation of *miR-3151* and *miR-203*, *-34b/c* or *34a* was studied by Chi-square test. The mean values of MTT assay, Trypan blue exclusion assay and cell apoptosis assay in WAC3CD5+ cells transfected with precursor *miR-3151* mimic (*MADD* or *PIK3R2* siRNA) were compared with negative control transfected with a scrambled oligo (control siRNA) by Student's *t*-test. All *P* values were two-sided.

## SUPPLEMENTARY MATERIALS FIGURES AND TABLE


