# Supplementary material for: Epigenetic silencing of tumor suppressor miR-3151 contributes to Chinese chronic lymphocytic leukemia by constitutive activation of MADD/ERK and PIK3R2/AKT signaling pathways
Source: Oncotarget. 2015 Oct 27;6(42):44422–36. doi: 10.18632/oncotarget.6251 (PMC4792566; doi:10.18632/oncotarget.6251)
Supplement: Supplementary file 1 [file oncotarget-06-44422-s001.pdf]

Epigenetic silencing of tumor suppressor *miR-3151* contributes to Chinese chronic lymphocytic leukemia by constitutive activation of MADD/ERK and PIK3R2/AKT signaling pathways

Supplementary Materials

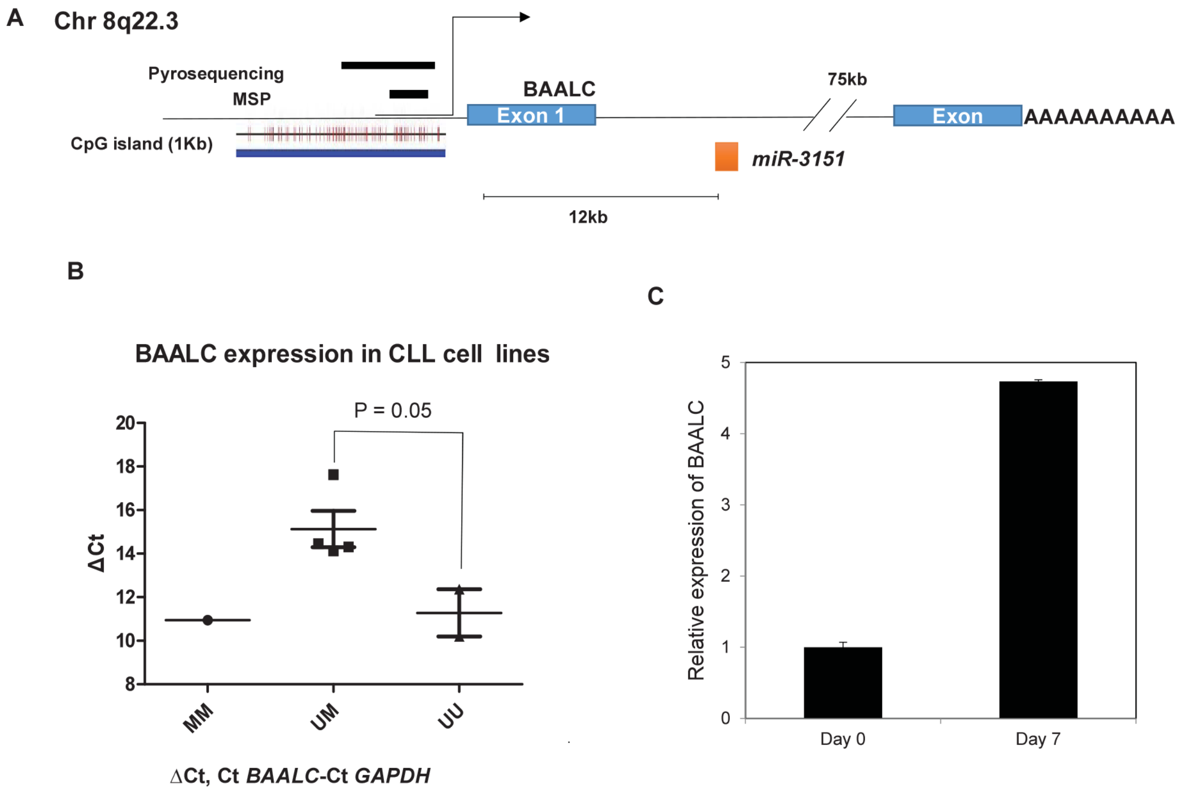

Supplementary Figure S1: Schematic diagram of *miR-3151* and its host gene *BAALC* and *BAALC* expression in CLL cell lines. (**A**) Schematic diagram of *miR-3151* and its host gene *BAALC*. It showed the distribution of CpG dinucleotides (solid vertical lines) at the promoter region of *miR-3151* and *BAALC*. Black bars indicated the amplification regions covered by MSP and pyrosequencing primers. (**B**) Real-time PCR analysis of *BAALC* expression in *BAALC* completely methylated, partially methylated and completely unmethylated CLL cell lines. ΔCt, Ct *BAALC*-Ct *GAPDH*. (**C**) Real-time-PCR analysis of *BAALC* expression in WAC3CD5+ cells before and after treatment with 0.5 µM 5-AzadC for 7 days. ΔCt, Ct *BAALC*-Ct *GAPDH*. *GAPDH* was used as reference for data analysis of *BAALC* expression by $2^{-\Delta\Delta CT}$ method.

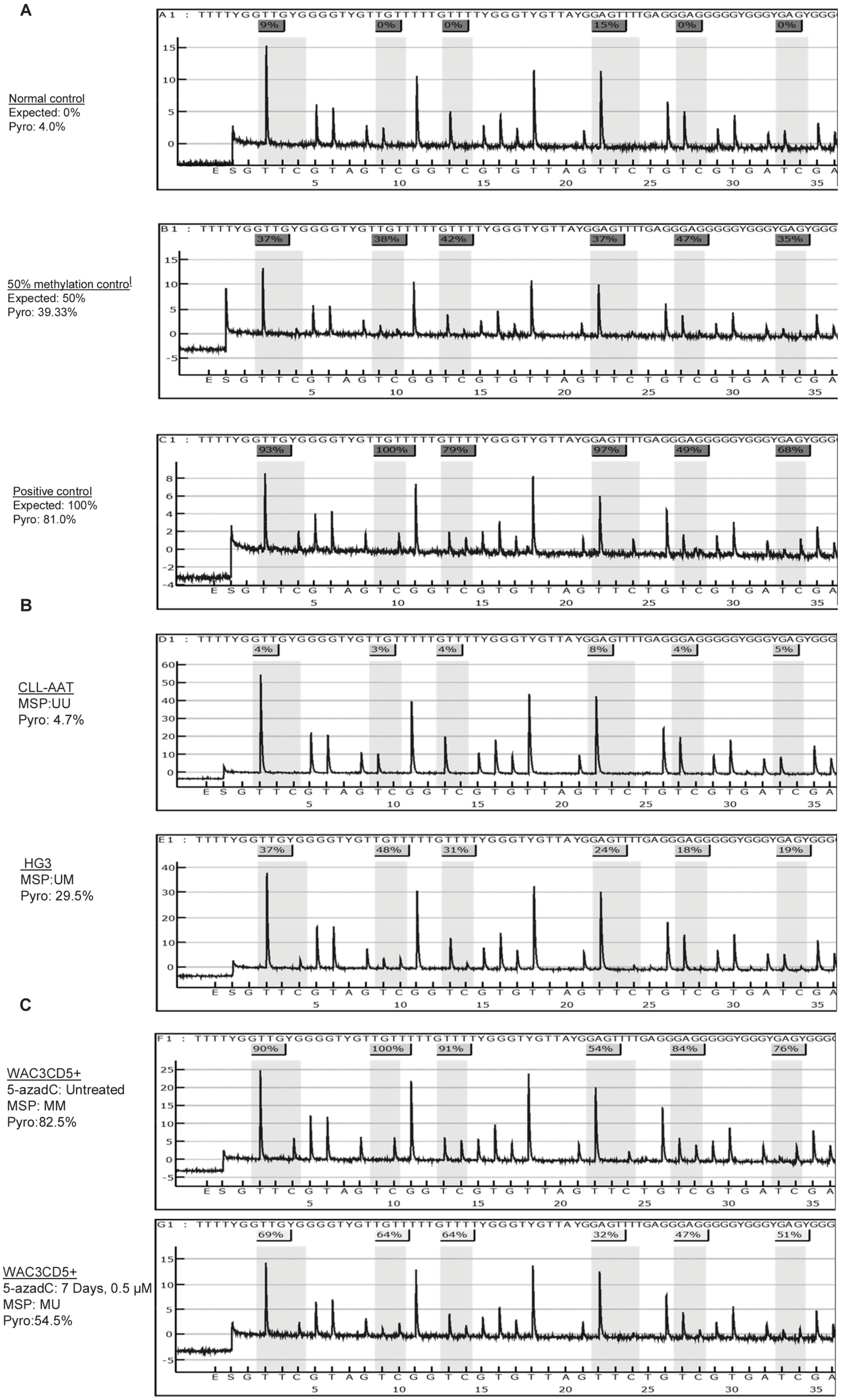

Supplementary Figure S2: Quantitative bisulfite pyrosequencing analysis of *miR-3151*. The pyrograms showed the methylation intensity on a stretch of 6 neighboring CpG dinucleotides of (**A**) Normal control without methylation, 50% methylation control and positive control with methylated DNA, (**B**) CLL cell lines with defined MSP methylation status (MU and UU) and (**C**) WAC3CD5+ cells (MM) before and after 5-azadC treatment.

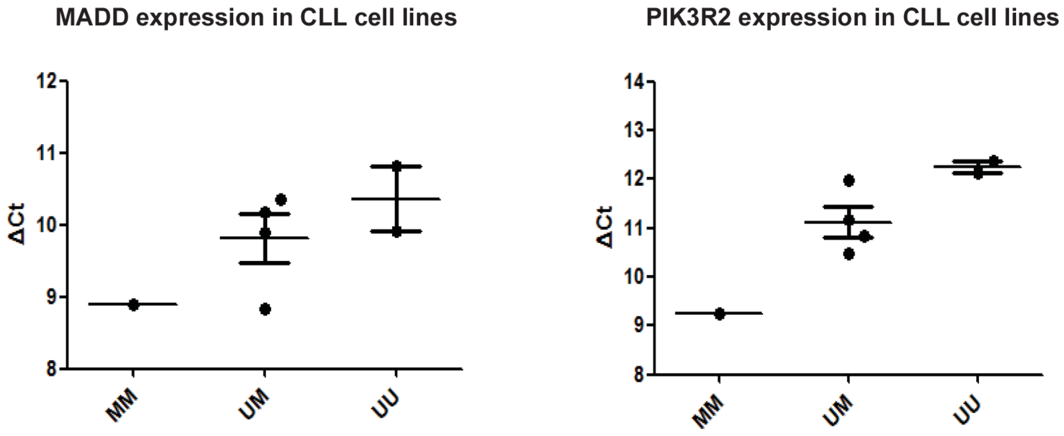

Supplementary Figure S3: *MADD* and *PIK3R2* expression in CLL cell lines. Real time-PCR analysis of *MADD* and *PIK3R2* expression in *miR-3151* completely methylated, partially methylated and completely unmethylated CLL cell lines. ΔCt, Ct *MADD/PIK3R2*-Ct *GAPDH*. *GAPDH* was used as reference for data analysis.

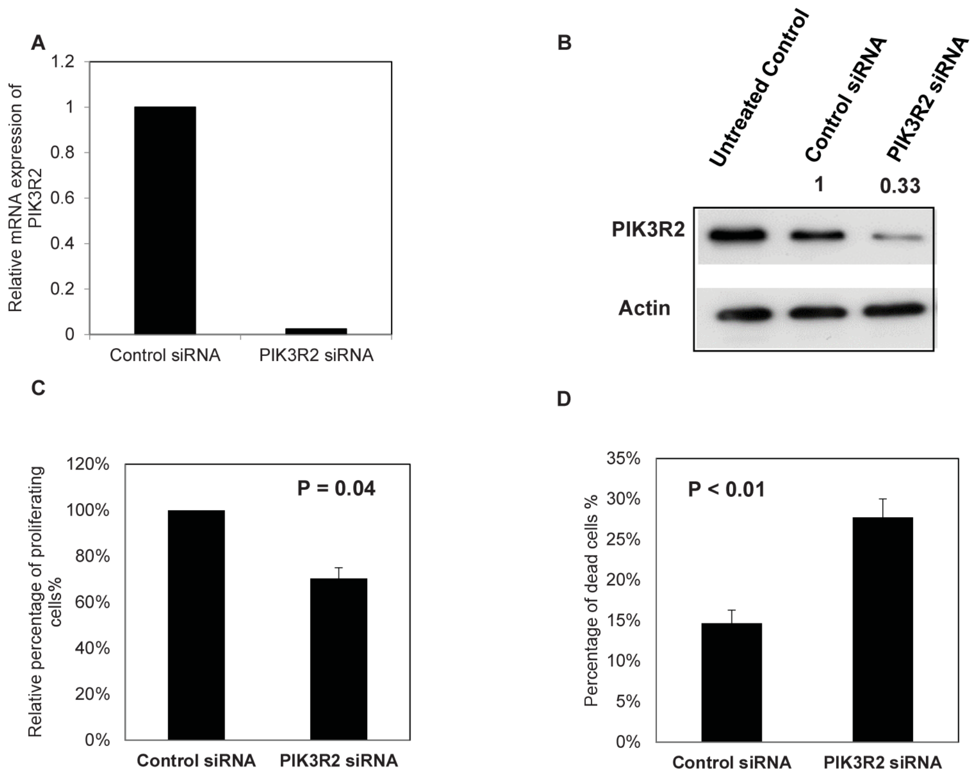

Supplementary Figure S4: Knockdown of *PIK3R2* in WAC3CD5+ cells. WAC3CD5+ cells, completely methylated for *miR-3151*, were transfected with control siRNA and *PIK3R2*-siRNA. Cells were harvested for further analysis 72 hours after transfection. Efficiency of *PIK3R2* knockdown by siRNA was shown at both mRNA and protein levels. (**A**) qRT-PCR showed the downregulation of *PIK3R2* upon *PIK3R2*-siRNA transfection in WAC3CD5+ cells as compared to control siRNA. ΔCt, Ct *PIK3R2*-Ct GAPDH. *GAPDH* was used as reference for data analysis of *PIK3R2* expression by $2^{-\Delta\Delta CT}$ method. (**B**) Western blot showed that knockdown of *PIK3R2*-siRNA led to reduction of *PIK3R2* protein. Actin was used as the endogenous control. The numbers indicated the relative protein expression measured by densitometric analysis. (**C**) Relative cell proliferation of CLL cells upon *PIK3R2*-siRNA knockdown was measured by MTT assay. Column, mean relative percentage of proliferating cells from three transfection experiments with triplicate in each. (**D**) Percentage of dead cells was measured by Trypan blue exclusion assay. Column, mean percentage of dead cells from three transfection experiments with triplicate in each. Error bars represent standard deviation.

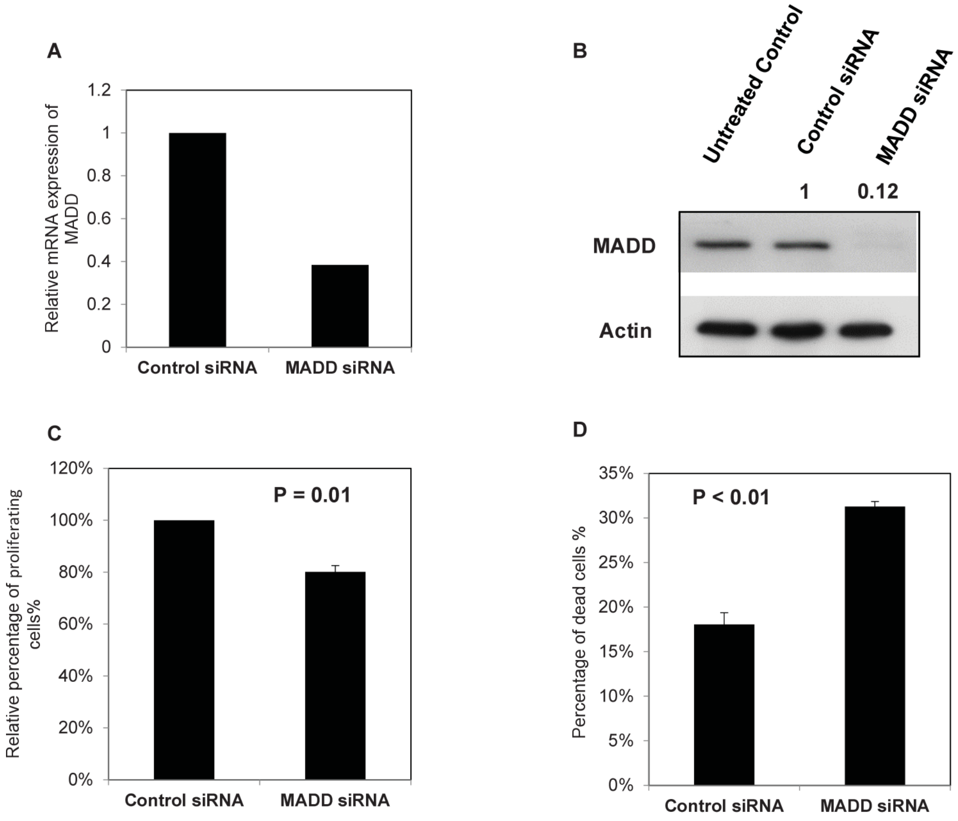

Supplementary Figure S5: Knockdown of *MADD* in WAC3CD5+ cells. WAC3CD5+ cells were transfected with control siRNA and *MADD*-siRNA. Cells were harvested for further analysis 48 hours after transfection. Efficiency of *MADD* knockdown by siRNA was shown at both mRNA and protein levels. (**A**) Knockdown of *MADD* with siRNA led to the reduction of *MADD* at mRNA level. ΔCt, Ct *MADD*-Ct *GAPDH*. *GAPDH* was used as reference for data analysis of *MADD* expression by $2^{-\Delta\Delta CT}$ method. (**B**) Western blot revealed that knockdown with *MADD*-siRNA resulted in the reduction of MADD protein. Actin was used as the endogenous control. The numbers indicated the relative protein expression measured by densitometric analysis. (**C**) Relative cell proliferating cells and (**D**) Percentage of dead cells upon *MADD*-siRNA knockdown was measured by MTT assay and Trypan blue exclusion assay respectively. Column, mean relative percentage of proliferating cells or mean percentage of dead cells from three transfection experiments with triplicate in each. Error bars represent standard deviation.

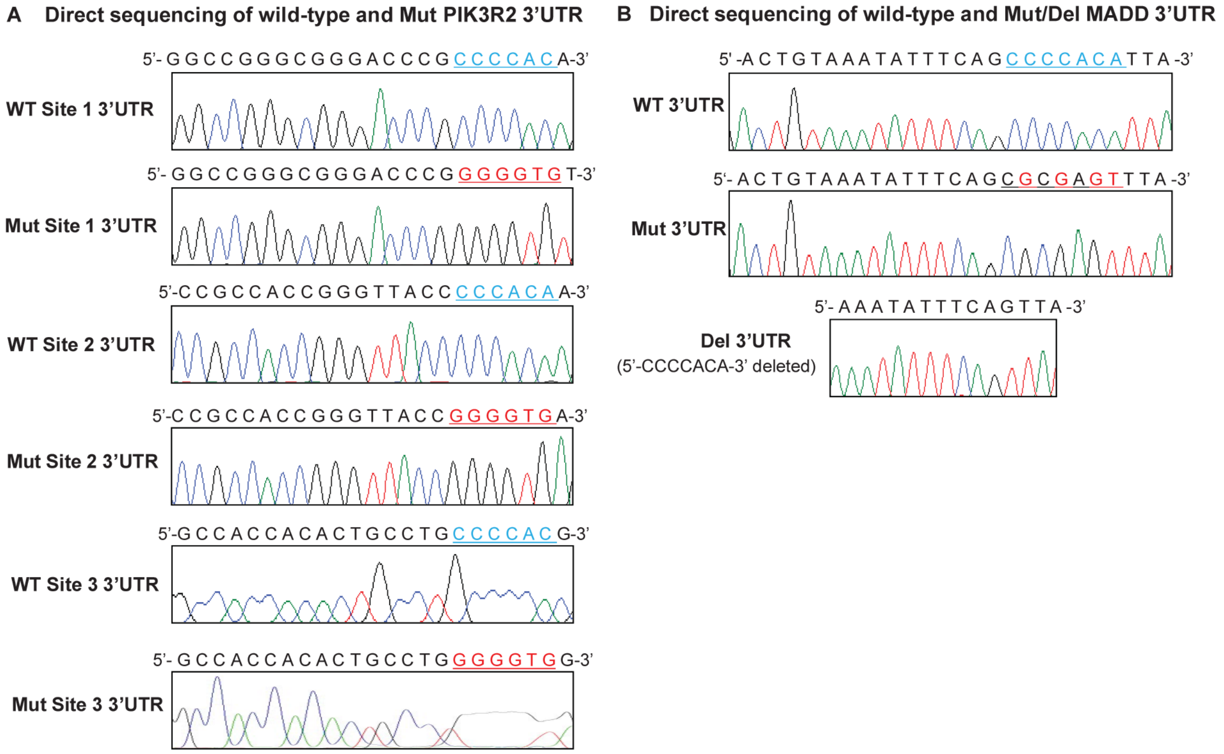

Supplementary Figure S6: Direct sequencing of wild-type and mutant 3′UTR of *PIK3R2* and *MADD*. (**A**) Sequence analysis of wild-type and Mut *PIK3R2*-3′UTR. The latter one contained a mutated S1, S2 and S3 with six point mutations inserted simultaneously. (**B**) The sequence of Mut *MADD*-3'UTR revealed 4 point mutation introduced in the seed region binding site (Mut *MADD*-3′UTR) while the other without all 7 bp (5′-CCCCACA-3′) of miRNA-mRNA interaction (Del *MADD*-3′UTR). The wild-type nucleotides in the 3′UTR of *PIK3R2* or *MADD* are indicated in blue and the corresponding mutated bases are in red.

Supplementary Table S1: RT-PCR, 3′UTR plasmid construct (Wild-type and mutant) primer sequences of *MADD* and *PIK3R2*

Gene	Forward primer (5′ to 3′)	Reverse primer (5′ to 3′)
***MADD* mRNA**	GTCCACACCGTCCACTGAAT	AGTCCTGGCCAATTTCTCGG
***PIK3R2* mRNA**	CAGTCCTGGCCAATGGAGG	AACGGAGCAGAAGGTGAGTG
**Wild-type *MADD* 3′UTR**	CTAGCTAGCCAGAGGATGGAGTGGTC	ACGCGTCGACACACAACTGTACATTTTCTAT
**Wild-type *PIK3R2* 3′UTR**	CTAGCTAGCAACTTCACTCTGCTGCTTCC	ACGCGTCGACGTACCGTACAAAACCTGCCT
**Mut *MADD* 3′UTR**	GGCAAGAAATGACTGTAAATATTTCAGCGCG AGTTTATTTATAGAAAATGTACAGTTGTGTG	CACACAACTGTACATTTTCTATAAATAA ACTCGCGCTGAAATATTTACAGTCATTT ACTCGCGCTGAAATATTTA
**Del *MADD* 3′UTR**	GGCAAGAAATGACTGTAAATATTTCAGTTA TTTATAGAAAATGTACAGTTGTGT	ACACAACTGTACATTTTCTATAAATAACT GAAATATTTACAGTCATTTCTTGCC
**Mut *PIK3R2* 3′UTR**	Site 1:5′-GGGCCGGGCGGGACCCGGGGGTGTGACTCCAACTTCCCCTC-3′<br>Site 2:5′-CCGCCACCGGGTTACCGGGGTGAGGGGGCCGCTGCGAG-3′<br>Site 3:5′-GGCCACCACACTGCCTGGGGGTGGAGCTGGGAGGCAGGTT-3′	
